# PD-1 inhibitors plus anti-angiogenic therapy with or without intensity-modulated radiotherapy for advanced hepatocellular carcinoma: A propensity score matching study

**DOI:** 10.3389/fimmu.2022.972503

**Published:** 2022-09-23

**Authors:** Ke Su, Lu Guo, Wenqiong Ma, Jing Wang, Yunchuan Xie, Mingyue Rao, Jianwen Zhang, Xueting Li, Lianbin Wen, Bo Li, Xiaoli Yang, Yanqiong Song, Weihong Huang, Hao Chi, Tao Gu, Ke Xu, Yanlin Liu, Jiali Chen, Zhenying Wu, Yi Jiang, Han Li, Hao Zeng, Pan Wang, Xunjie Feng, Siyu Chen, Binbin Yang, Hongping Jin, Kun He, Yunwei Han

**Affiliations:** ^1^ Department of Oncology, The Affiliated Hospital of Southwest Medical University, Luzhou, China; ^2^ Department of Ophthalmology, The Affiliated Hospital of Southwest Medical University, Luzhou, China; ^3^ Clinical Research Institute, The Affiliated Hospital of Southwest Medical University, Luzhou, China; ^4^ Department of Radiology, The Affiliated Hospital of Southwest Medical University, Luzhou, China; ^5^ Department of Oncology, 363 Hospital, Chengdu, China; ^6^ Department of Geriatric Cardiology, Sichuan Academy of Medical Sciences & Sichuan Provincial People’s Hospital, Chengdu, China; ^7^ Department of General Surgery (Hepatobiliary Surgery), The Affiliated Hospital of Southwest Medical University, Luzhou, China; ^8^ Nuclear Medicine and Molecular Imaging Key Laboratory of Sichuan Province, Luzhou, China; ^9^ Academician (Expert) Workstation of Sichuan Province, Luzhou, China; ^10^ Department of Radiotherapy, Sichuan Cancer Hospital & Institute, Sichuan Cancer Center, School of Medicine, University of Electronic Science and Technology of China, Chengdu, China; ^11^ Clinical Medical College, Southwest Medical University, Luzhou, China; ^12^ Clinical Skills Center, The Affiliated Hospital of Southwest Medical University, Luzhou, China

**Keywords:** programmed death-1 inhibitors, anti-angiogenic therapy, intensity-modulated radiotherapy, hepatocellular carcinoma, propensity score matching

## Abstract

**Background:**

Whether intensity-modulated radiotherapy (IMRT) can enhance the efficacy of the programmed death (PD)-1 inhibitors combined with anti-angiogenic therapy for hepatocellular carcinoma (HCC) is unclear. Therefore, we conducted this multicenter retrospective study to investigate the efficacy of the combination of PD-1 inhibitors with anti-angiogenic therapy and IMRT.

**Methods:**

From April 2019 to March 2022, a total of 197 patients with HCC [combination of PD-1 inhibitors with anti-angiogenic therapy and IMRT (triple therapy group), 54; PD-1 inhibitors plus anti-angiogenic therapy (control group), 143] were included in our study. Propensity score matching (PSM) was applied to identify two groups with similar baselines. The objective response rate (ORR), overall survival (OS), and progression-free survival (PFS) of the two groups were compared before and after matching.

**Results:**

Prior to PSM, the triple therapy group had higher ORR (42.6% vs 24.5%, *P* = 0.013) and more superior median OS (mOS) (20.1 vs 13.3 months, *P* = 0.009) and median PFS (mPFS) (8.7 vs 5.4 months, *P* = 0.001) than the control group. Following PSM, the triple therapy group still exhibited better mPFS (8.7 vs 5.4 months, *P* = 0.013) and mOS (18.5 vs 12.6 months, *P* = 0.043) than the control group. However, the ORR of the two groups was similar (40% vs 25%, *P* = 0.152). No significant difference was observed in the treatment-related adverse events between the two groups (*P* < 0.05 for all).

**Conclusions:**

The combination of PD-1 inhibitors with anti-angiogenic therapy and IMRT for HCC is a promising regimen.

## Introduction

Hepatocellular carcinoma (HCC) is the most common cause of cancer-related death (1). Despite the wide use of early detection techniques to diagnose HCC, most patients are diagnosed at an advanced stage (2). The overall survival (OS) of patients with HCC is extremely short, therefore, the prognosis of patients should be urgently improved (3).

Currently, the combination of programmed death 1/programmed death ligand 1 (PD-1/PD-L1) inhibitors and targeted drugs has become prominent in HCC research. Atezolizumab plus bevacizumab, the current first-line treatment option, extends median OS (mOS) to 19.2 months and objective response rate (ORR) to 27.3% in inoperable HCC (4, 5). Additionally, Ren et al. (6) reported an ORR of 21% and a median progression-free survival (mPFS) of 4.6 months in patients with inoperable HCC who received sintilimab plus bevacizumab. In the RESCUE study of camrelizumab plus apatinib for advanced HCC, the ORR was 34.3% and mPFS was 5.7 months (7). Despite breakthroughs in the combination therapy of PD-1/PD-L1 inhibitors and targeted drugs, its ORR was still low. The addition of other treatments that can improve local control of HCC has become a new research direction.

Intensity-modulated radiotherapy (IMRT), an external RT modality, is a local treatment method that uses radiation to irradiate malignant tumor cells. Abulimiti et al. (8) confirmed that IMRT plus sorafenib can improve the prognosis of advanced HCC, for which the mOS was observed to be 11.4 months and the mPFS was 6 months. Additionally, patients with advanced HCC who received IMRT in combination with apatinib had an mPFS of 7.8 months and an ORR of 15% (9). Radiotherapy can not only promote the generation and infiltration of T cells but also stimulate systemic anti-tumor immunity to control metastatic lesions, causing the “abscopal effect” (10). Furthermore, targeting vascular endothelial growth factor (VEGF) can normalize tumor vessels and enhance T cell infiltration, thus, providing a rationale for combining this therapy with immunotherapy (11).

Based on these results, the combination of PD-1 inhibitors with anti-angiogenic therapy and IMRT is a promising treatment modality. We conducted this multicenter retrospective study to investigate the efficacy of triple therapy.

## Materials and methods

### Patients

From April 2019 to March 2022, a total of 197 patients with HCC [combination of PD-1 inhibitors with anti-angiogenic therapy and IMRT (triple therapy group), 54; PD-1 inhibitors plus anti-angiogenic therapy (control group), 143] from three Chinese tertiary hospitals were included in our retrospective study.

The inclusion criteria were as follows: a) Pathologically diagnosed HCC; b) Barcelona Clinic Liver Cancer (BCLC) stage B/C; c) Eastern Cooperative Oncology Group performance status (ECOG PS) score of 0−2; d) Child-Pugh class A/B; e) at least one measurable lesion according to the modified Response Evaluation Criteria in Solid Tumors (mRECIST); f) administration of at least one cycle of PD-1 inhibitors plus anti-angiogenic therapy with or without IMRT; g) patients were able to undergo IMRT after evaluation. The exclusion criteria were as follows: a) Incomplete information; b) number of tumors >5 or diffuse lesions; c) presence of other malignancies; d) severe ascites or hepatic encephalopathy.

This study was approved by the Ethics Committee of the affiliated hospital of Southwest Medical University (approval number KY2020254). We waived individual informed consent since this was a retrospective study.

### Treatment protocol

#### Imrt

IMRT was performed within 7 days of the administration of the first cycle of PD-1 inhibitors plus anti-angiogenic therapy. The radiologist used the radiation planning system to delineate the target volume with computed tomography (CT) guidance. Delineation of the clinical target volume (CTV) including a 4-mm margin of the primary liver tumor was accomplished through image technology. The planning target volume (PTV) was defined as a 5-10-cm peripheral expansion based on CTV. The total target radiation dose was 48 g with 3 Gy/fraction, and at least 95% of PTV received the prescribed dose. The dose constraints for the organs at risk were as follows: Spinal cord (maximum dose ≤45 Gy); normal liver (mean dose ≤30 Gy); stomach and duodenum (maximum dose ≤54 Gy); colon (maximum dose ≤55 Gy).

### Administration of PD-1 inhibitors and targeted agents

All patients received PD-1 inhibitor injection once every three weeks as well as the antiangiogenic drug on daily basis until the appearance of intolerable toxic reactions or progressive disease. The doses of PD-1 inhibitors and targeted drugs were calculated based on the patient’s height and weight. Dosing delays were allowed when a serious treatment-related adverse event (TRAE) occurred.

### Follow-up and data collection

The efficacy of patients was assessed by CT/Magnetic Resonance Imaging (MRI) performed every 2−3 months. Treatment response was divided into complete response (CR), partial response (PR), stable disease (SD), and progressive disease according to mRECIST. The time interval from treatment initiation to progressive disease was PFS. The time interval from the initiation of treatment to the death or last follow-up was OS.

### Statistical analysis

χ^2^ test and McNemar analysis were used for categorical variables. Propensity score matching (PSM) was applied to identify two groups with similar baselines. Matching variables included age, sex, tumor size, alanine transaminase level, tumor number, platelet level, alkaline phosphatase level, Child-Pugh score, alpha-fetoprotein (AFP) level, leukocyte level, BCLC stage, portal vein invasion, hepatitis B virus infection, extrahepatic metastasis, and lymph node metastasis. PFS and OS were estimated using the Kaplan–Meier method and log-rank test. Cox analysis was used to identify prognostic factors affecting OS and PFS. Statistical analysis of this study was performed using SPSS for Windows version 26.0. Two-tailed *P*-value of <0.05 was considered significant.

## Results

### Patient characteristics prior to and following PSM

Between April 2019 and March 2022, a total of 197 patients who met the inclusion and exclusion criteria received the combination of PD-1 inhibitors with anti-angiogenic therapy and IMRT and PD-1 inhibitors plus anti-angiogenic therapy.

Prior to PSM, there were differences in gender, leukocyte level, BCLC stage, lymph node metastasis, and extrahepatic metastases between the two groups (*P* < 0.05 for all). Eighty patients were identified through PSM. In this matched cohort, no differences in any covariates at baseline were observed between the two groups (**Table 1**).

**Table 1:** Baseline characteristics of the patients before and after PSM.

Variable	Before PSM			After PSM		
	Triple therapy group	Control group	*P*	Triple therapy group	Control group	*P*
Patients	54	143		40	40	
Male sex	51 (94.4)	112 (78.3)	0.008	37 (92.5)	38 (95.0)	1.000
Age ≥ 65 years	11 (20.4)	29 (20.3)	0.989	10 (25.0)	6 (15.0)	0.424
Child–Pugh score			0.735			0.568
5	20 (37.0)	62 (43.4)		16 (40.0)	15 (37.5)	
6	20 (37.0)	39 (27.3)		13 (32.5)	13 (32.5)	
7	8 (14.8)	27 (18.9)		6 (15.0)	8 (20.0)	
8	4 (7.4)	10 (7.0)		3 (7.5)	2 (5.0)	
9	2 (3.7)	5 (3.5)		2 (5.0)	2 (5.0)	
Number of tumors ≥ 2	40 (74.1)	118 (82.5)	0.185	31 (77.5)	28 (70.0)	0.629
Tumor diameter, cm			0.243			0.937
< 3	3 (5.6)	9 (6.3)		2 (5.0)	1 (2.5)	
≥ 3, < 5	6 (11.1)	34 (23.8)		6 (15.0)	6 (15.0)	
≥ 5, < 10	30 (55.6)	69 (48.3)		21 (52.5)	20 (50.0)	
≥ 10	15 (27.8)	31 (21.7)		11 (27.5)	13 (32.5)	
Serum AFP, ng/ml			0.700			0.572
< 200	27 (50.0)	79 (55.2)		21 (52.5)	17 (42.5)	
≥ 200, < 400	2 (3.7)	7 (4.9)		2 (5.0)	4 (10.0)	
≥ 400	25 (46.3)	57 (39.9)		17 (42.5)	19 (47.5)	
ALP levels ≥ 125 U/L	26 (48.1)	87 (60.8)	0.108	23 (57.5)	23 (57.5)	1.000
Platelet count ≥ 100 × 109/L	46 (85.2)	109 (76.2)	0.171	32 (80.0)	35 (87.5)	0.581
ALT levels ≥ 40 U/L	31 (57.4)	74 (51.7)	0.478	20 (50.0)	20 (50.0)	1.000
Leukocyte ≥ 4 × 109/L	41 (75.9)	128 (89.5)	0.015	30 (75.0)	34 (85.0)	0.388
BCLC stage			0.041			1.000
B	3 (5.6)	24 (16.8)		3 (7.5)	3 (7.5)	
C	51 (94.4)	119 (83.2)		37 (92.5)	37 (92.5)	
Portal vein invasion	46 (85.2)	91 (63.6)	0.003	32 (80.0)	32 (80.0)	1.000
HBV	33 (61.1)	77 (53.8)	0.360	24 (60.0)	21 (52.5)	0.678
Lymph node metastasis	21 (38.9)	80 (55.9)	0.033	19 (47.5)	19 (47.5)	1.000
Extrahepatic metastases	11 (20.4)	59 (41.3)	0.006	9 (22.5)	11 (27.5)	0.774
Lung	4 (7.4)	33 (23.1)		3 (7.5)	6 (15.0)	
Bone	6 (11.1)	15 (10.5)		5 (12.5)	4 (10.0)	
Other	1 (1.9)	28 (19.6)		1 (2.5)	5 (12.5)	

PSM, propensity score matching; AFP, alpha fetoprotein; ALP, alkaline phosphatase; ALT, alanine aminotransferase; BCLC, Barcelona Clinic Liver Cancer; HBV, hepatitis B virus.

### The triple therapy group exhibited promising efficacy

**Figure 1:**
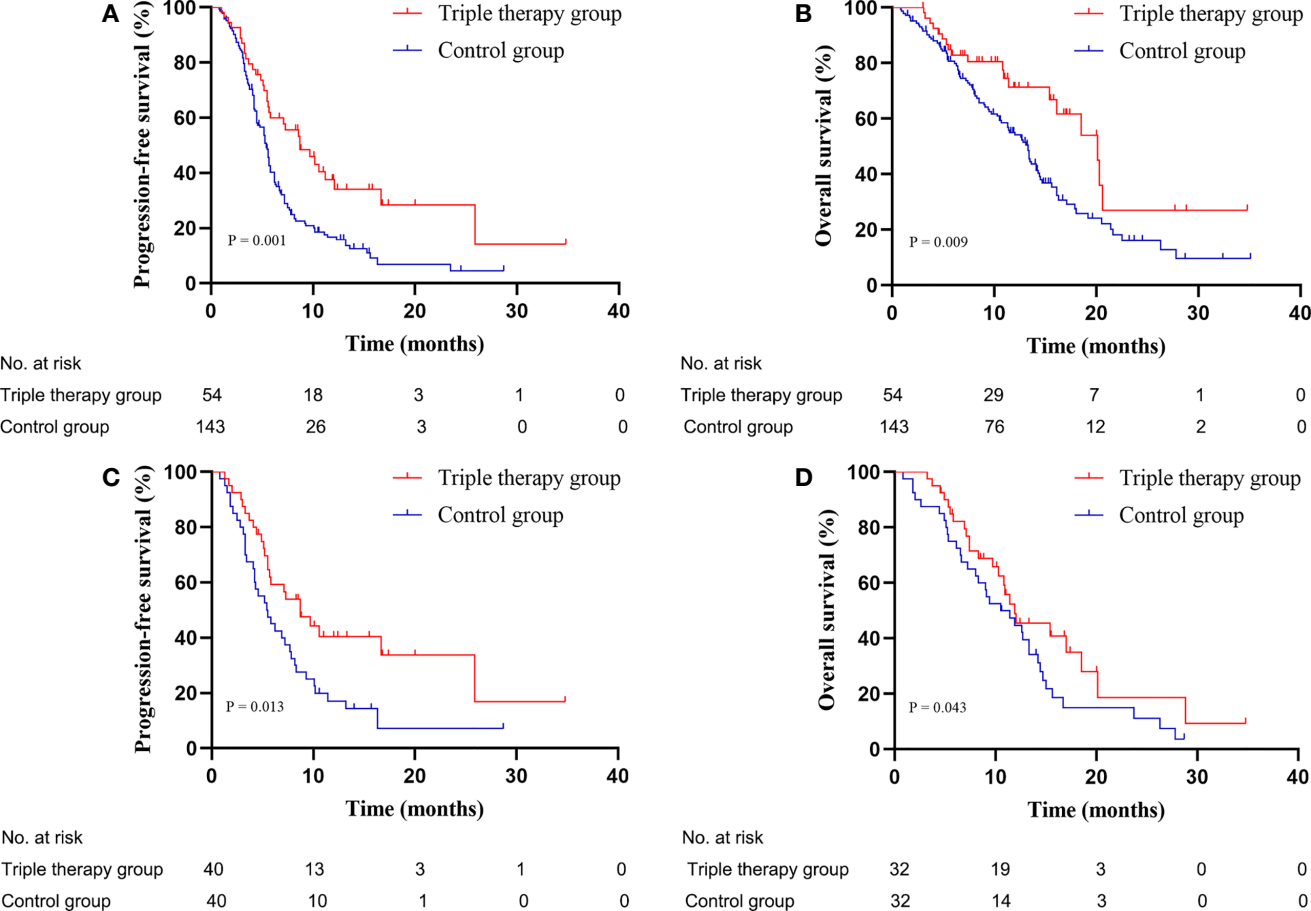
Kaplan-Meier plots: The triple therapy group exhibited longer mPFS **(A, C)** and mOS **(B, D)** than that of the control group before and after PSM. mPFS, median progression-free survival; mOS, median overall survival; PSM, propensity score matching.

As of April 2022, before matching, a total of 91 (63.6%) and 19 (35.2%) patients died in the control group and the triple therapy group, respectively. The median follow-up time of the control group and triple therapy group was 15.5 and 12 months, respectively. Patients who received triple therapy had longer mPFS (8.7 vs 5.4 months, *P* = 0.001, **Figure 1A**) and mOS (20.1 vs 13.3 months, *P* = 0.009, **Figure 1B**) than those who received PD-1 inhibitors plus anti-angiogenic therapy. Following PSM, 14 patients (35%) in the triple therapy group and 27 patients (67.5%) in the control group died. Patients who received triple therapy had longer mPFS (8.7 vs 5.4 months, *P* = 0.013, **Figure 1C**) and mOS (18.5 vs 12.6 months, *P* = 0.043, **Figure 1D**) than those who received PD-1 inhibitors plus anti-angiogenic therapy.

### PFS and OS in different subgroups

In the subgroup of patients with child-pugh class A and tumor diameter of ≥ 5 cm, the triple therapy group had longer mOS (not reach vs 14.4 months, *P* = 0.042, **Supplementary Figure 1C**; 18.5 vs 11.4 months, *P* = 0.018, **Supplementary Figure 1E**) and mPFS (25.9 vs 5.5 months, *P* = 0.005, **Supplementary Figure 1H**; 8.7 vs 5.4 months, *P* = 0.009, **Supplementary Figure 1J**) than the control group. However, in the subgroup analysis of patients with portal vein tumor thrombus (PVTT), child B, and extrahepatic metastases, there were no significant differences in OS and PFS between the two groups (**Supplementary Figure 1**).

### Tumor response

Prior to PSM, the ORR was 42.6% in the triple therapy group and 24.5% in the control group (*P* = 0.013). However, the disease control rates (DCR) of two groups were similar (90.7% vs 79.7%, *P* = 0.068). Following PSM, although the ORR and DCR of the triple therapy group were still slightly better than those of the control group, the differences were not significant (40% vs 25%, *P* = 0.152; 90% vs 77.5%, *P* = 0.130; respectively; **Table 2**).

**Table 2:** Tumor response assessed by mRECIST.

Best response	Before PSM			After PSM		
	Triple therapy group	Control group	*P*	Triple therapy group	Control group	*P*
Objective response	23 (42.6)	35 (24.5)	0.013	16 (40.0)	10 (25.0)	0.152
Disease control	49 (90.7)	114 (79.7)	0.068	36 (90.0)	31 (77.5)	0.130
Best overall response						
Complete response	1 (1.9)	2 (1.4)		1 (2.5)	0	
Partial response	22 (40.7)	33 (23.1)		15 (37.5)	10 (25.0)	
Stable disease	26 (48.1)	79 (55.2)		20 (50.0)	21 (52.5)	
Progressive disease	5 (9.3)	29 (20.3)		4 (10.0)	9 (22.5)	

mRECIST, modified Response Evaluation Criteria in Solid Tumors.

### Factors associated with PFS and OS following PSM

Univariate and multivariate Cox regression analyses were used to identify prognostic indicators affecting PFS and OS following PSM. Age, Child-Pugh class, AFP level, and triple therapy were determined to be influencing factors for PFS and OS (*P* < 0.05 for all). In the multivariate analysis, an AFP level of ≥400 ng/mL was an independent negative prognostic factor for PFS (**Table 3**), whereas child B, lymph node metastasis, and treatment method were independent prognostic factors for OS (**Table 4**).

**Table 3:** Univariate and multivariate Cox regression analysis of progression-free survival after PSM.

Variable	Univariable Cox regression			Multivariable Cox regression		
	HR	95%CI	*P*	HR	95%CI	*P*
Sex (male/female)	2.121	0.657-6.853	0.209			
Age (≥65/<65 years)	0.366	0.172-0.779	0.009	0.481	0.220-1.052	0.067
Child-Pugh class (B/A)	2.109	1.227-3.623	0.007	1.564	0.892-2.74	0.118
Number of tumors (≥2/<2)	1.584	0.859-2.922	0.141			
Tumor diameter (≥5/<5 cm)	1.334	0.672-2.648	0.409			
AFP (≥400/<400 ng/ml)	2.86	1.676-4.878	<0.001	2.043	1.158-3.605	0.014
ALP (≥125/<125 U/L)	1.202	0.716-2.016	0.487			
Platelet (<100000/≥100000/μL)	0.798	0.422-1.507	0.487			
ALT (≥40/<40U/L)	1.129	0.676-1.887	0.642			
Leukocyte (<4000/≥4000/μL)	0.730	0.400-1.333	0.305			
HBV (positive/negative)	1.044	0.622-1.751	0.870			
Portal vein invasion (yes/no)	1.291	0.669-2.490	0.446			
BCLC stage (C/B)	1.008	0.363-2.798	0.987			
Lymph node metastasis (yes/no)	1.287	0.767-2.159	0.340			
Extrahepatic metastases (yes/no)	1.090	0.605-1.962	0.774			
Triple therapy (Yes/No)	0.522	0.309-0.882	0.015	0.603	0.354-1.029	0.063

PSM, propensity score matching; HR, hazard ratio; AFP, alpha fetoprotein; ALP, alkaline phosphatase; ALT, alanine transaminase; HBV, hepatitis B virus; BCLC, Barcelona Clinic Liver Cancer.

**Table 4:** Univariate and multivariate Cox regression analysis of overall survival after PSM.

Variable	Univariable Cox regression			Multivariable Cox regression		
	HR	95%CI	*P*	HR	95%CI	*P*
Sex (male/female)	1.924	0.460-8.048	0.37			
Age (≥65/<65 years)	0.364	0.142-0.934	0.036	0.460	0.170-1.242	0.125
Child-Pugh class (B/A)	3.638	1.919-6.897	<0.001	3.114	1.538-6.305	0.002
Number of tumors (≥2/<2)	2.035	0.931-4.449	0.075			
Tumor diameter (≥5/<5 cm)	1.334	0.605-2.939	0.475			
AFP (≥400/<400 ng/ml)	2.539	1.344-4.797	0.004	1.856	0.919-3.748	0.084
ALP (≥125/<125 U/L)	1.300	0.693-2.439	0.413			
Platelet (<100000/≥100000/μL)	0.877	0.400-1.924	0.744			
ALT (≥40/<40U/L)	0.927	0.501-1.716	0.809			
Leukocyte (<4000/≥4000/μL)	0.72	0.343-1.511	0.385			
HBV (positive/negative)	1.017	0.545-1.899	0.957			
Portal vein invasion (yes/no)	2.091	0.819-5.339	0.123			
BCLC stage (C/B)	3.172	0.434-23.157	0.255			
Lymph node metastasis (yes/no)	1.928	1.014-3.665	0.045	2.002	1.036-3.871	0.039
Extrahepatic metastases (yes/no)	0.963	0.470-1.975	0.919			
Triple therapy (Yes/No)	0.520	0.272-0.993	0.048	0.511	0.262-0.996	0.049

PSM, propensity score matching; HR, hazard ratio; AFP, alpha fetoprotein; ALP, alkaline phosphatase; ALT, alanine transaminase; HBV, hepatitis B virus; BCLC, Barcelona Clinic Liver Cancer.

### Safety

We further investigated the TRAEs of the two groups. Treatment was interrupted in 55 patients (triple therapy group, 18; control group, 37) secondary to serious TRAEs. The addition of IMRT did not significantly increase the TRAEs of PD-1 inhibitors plus anti-angiogenic therapy (*P* < 0.05 for all). There were no treatment-related deaths (**Table 5**).

**Table 5:** Treatment-related adverse events in the two groups.

Adverse Event	Triple therapy group		Control group		
	Grade 1-2	Grade ≥3	Grade 1-2	Grade ≥3	*P*
Leukopenia	29 (53.7)	4 (7.4)	58 (40.6)	8 (5.6)	0.173
Thrombocytopenia	24 (44.4)	3 (5.6)	52 (36.4)	7 (4.9)	0.541
Decreased appetite	15 (27.8)	3 (5.6)	32 (22.4)	7 (4.9)	0.699
Neutropenia	14 (25.9)	1 (1.9)	26 (18.2)	2 (1.4)	0.461
Fatigue	6 (11.1)	2 (3.7)	14 (9.8)	5 (3.5)	0.959
Nausea	8 (14.8)	3 (5.6)	16 (11.2)	5 (3.5)	0.612
Anemia	7 (13.0)	1 (1.9)	9 (6.3)	1 (0.7)	0.232
Increased alanine aminotransferase	10 (18.5)	2 (3.7)	18 (12.6)	1 (0.7)	0.160
Rash	4 (7.4)	2 (3.7)	8 (5.6)	1 (0.7)	0.268
Pruritus	4 (7.4)	0	9 (6.3)	1 (0.7)	0.798
Fever	3 (5.6)	0	5 (3.5)	0	0.514
Increased aspartate aminotransferase	9 (16.7)	2 (3.7)	14 (9.8)	3 (2.1)	0.314
Hypothyroidism	3 (5.6)	0	5 (3.5)	0	0.514
Hypertension	2 (3.7)	0	3 (2.1)	0	0.523
Headache	1 (1.9)	0	1 (0.7)	1 (0.7)	0.640

## Discussion

Currently, although atezolizumab plus bevacizumab is the first recommendation for treating advanced HCC, its ORR of 27.3% remains unsatisfactory (4, 5). Therefore, it is necessary to explore other therapeutic methods that can improve the local control of advanced HCC. This was the first study on PD-1 inhibitors with anti-angiogenic therapy and IMRT vs PD-1 inhibitors plus anti-angiogenic therapy for the treatment of advanced HCC.

Prior to PSM, the triple therapy group had higher ORR (42.6% vs 24.5%, *P* = 0.013) and longer mOS (20.1 vs 13.3 months, *P* = 0.009) and mPFS (8.7 vs 5.4 months, *P* = 0.001) than those of the control group. Following PSM, the triple therapy group revealed better efficacy than the control group. This may be owing to strong local control of radiotherapy (12, 13). It not only induces immunogenic death but also modulates the tumor microenvironment to stimulate the production of antitumor T cells (14, 15). Moreover, radiotherapy increases the production of cell adhesion molecules, and targeting VEGF can promote the normalization of the vascular endothelium. This further enhances antitumor T cell infiltration (11, 16, 17).

Currently, new techniques such as stable homogeneous iodinated formulation technology hold good potential for surgical resection after arterial embolization in clinical practice (18). However, many HCC patients have already lost the opportunity for surgery. Immunotherapy plus targeted therapy for advanced HCC has been the focus of research (4–7), whereas the research on the combination of radiotherapy and immunotherapy is in its infancy. In a retrospective study of patients with HCC receiving stereotactic body radiotherapy (SBRT) plus PD-1 inhibitors, the mPFS was 19.6 months and ORR was 71% (19). Zhong et al. (20) observed that patients with advanced HCC treated with SBRT combined with PD-1 inhibitors had a higher ORR of 40%, mPFS of 3.8 months, and mOS of 21.2 months. Additionally, Ricke et al. reported that the mOS of patients with HCC receiving selective internal radiation therapy plus sorafenib was 12.1 months (21). Further, satisfactory results were also obtained with nivolumab plus ipilimumab for advanced HCC (mOS = 22.8 months, ORR = 32%) (22). In our study, the triple therapy group revealed better efficacy than the control group.

The safety of other methods based on PD-1 inhibitors plus anti-angiogenic therapy has been questioned. Liu et al. (23) confirmed that patients with HCC treated with hepatic artery infusion chemotherapy, tyrosine kinase inhibitors, and anti-PD-1 antibodies exhibited good efficacy (mPFS = 10.6 months, ORR = 63%) and safety. Furthermore, among patients with unresectable HCC, transarterial chemoembolization-lenvatinib-pembrolizumab sequential therapy exhibited promising efficacy (mPFS = 9.2 months, mOS = 18.1 months), with a well-characterized safety profile (24). In our research, we confirmed that the addition of IMRT did not significantly increase the TRAEs of PD-1 inhibitors plus anti-angiogenic therapy. Based on these findings, combining radiotherapy with immune and targeted therapies is a promising combination modality.

In the subgroups of patients with child A and tumor diameter ≥5 cm, the triple therapy group had more superior mOS and mPFS than the control group. However, in the other subgroups, there were no significant differences in OS and PFS between the two groups. Additionally, we observed that the ORR of the triple therapy group prior to PSM was better than that of the control group (42.6% vs 24.5%, *P* = 0.013) whereas the ORR of the two groups of patients following PSM was similar (40% vs 25%, *P* = 0.152). These may be owing to the smaller sample size.

Further, we explored prognostic factors affecting PFS and OS. The AFP level of ≥400 ng/mL is a risk factor for disease progression. However, for child A, without lymph node metastasis, triple therapy was an independent prognostic factor causing longer OS. Moreover, previous studies have also reported that these indicators were associated with prognosis (25–27).

This study had some limitations. First, although PSM was performed to minimize the effects of observed confounding factors, the effects of selectivity bias and various potential defects were not excluded. Second, despite this being the largest study reported to date, the number of patients in the triple therapy group remained less. Last, although our study confirms that IMRT further improves the efficacy of the combination of PD-1 inhibitors and anti-angiogenic therapy, it is still affected by the underlying heterogeneity of different therapeutic agents.

## Conclusions

Conclusively, this study confirmed that the combination of PD-1 inhibitors with anti-angiogenic therapy and IMRT is a promising combination regimen. Our study provides a theoretical basis for studying combination therapy for HCC. Future prospective studies with larger sample sizes are needed to determine the efficacy of triple therapy.

## Data availability statement

The raw data supporting the conclusions of this article will be made available by the authors, without undue reservation.

## Ethics statement

The studies involving human participants were reviewed and approved by the Ethics Committee of the affiliated hospital of Southwest Medical University (approval number KY2020254). Written informed consent for participation was not required for this study in accordance with the national legislation and the institutional requirements.

## Author contributions

KS, LG, WM, JW, YX, MR, JZ, XL, LW, BL, XY, YS, WH, HC, TG, KX, YL, JC, ZW, YJ, HL, HZ, PW, XF, SC, BY, HJ, KH, and YH collected the data. YH and KH designed the research study. KS, LG, WM, and YH wrote the manuscript and analyzed the data. All authors approved the final version of the manuscript.

## Funding

This work was supported by the Project of Science and Technology Department of Sichuan Province (2020JDTD0036) and the Nuclear Medicine and Molecular Imaging Key Laboratory of Sichuan Province (HYX18001).

## Conflict of interest

The authors declare that the research was conducted in the absence of any commercial or financial relationships that could be construed as a potential conflict of interest.

## Publisher’s note

All claims expressed in this article are solely those of the authors and do not necessarily represent those of their affiliated organizations, or those of the publisher, the editors and the reviewers. Any product that may be evaluated in this article, or claim that may be made by its manufacturer, is not guaranteed or endorsed by the publisher.
