# Supplementary material for: PD-1 inhibitors plus anti-angiogenic therapy with or without intensity-modulated radiotherapy for advanced hepatocellular carcinoma: A propensity score matching study
Source: Front Immunol. 2022 Sep 23;13:972503. doi: 10.3389/fimmu.2022.972503 (PMC9539675; doi:10.3389/fimmu.2022.972503)
Supplement: Supplementary file 1 [file Image_1.pdf]

Supplementary Fig.1. Kaplan-Meier plots: Overall survival curves for the patients with portal vein tumor thrombus (A), extrahepatic metastases (B), child A (C), child B (D), tumor diameter ≥ 5 cm (E). Progression-free survival curves for the patients with portal vein tumor thrombus (F), extrahepatic metastases (G), child A (H), child B (I), tumor diameter ≥ 5 cm (J).

A
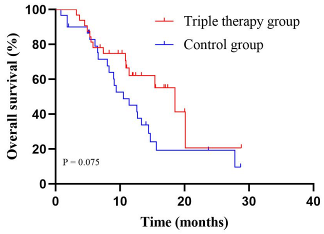

No. at risk					
Triple therapy group	32	19	3	0	0
Control group	30	14	3	0	0

B
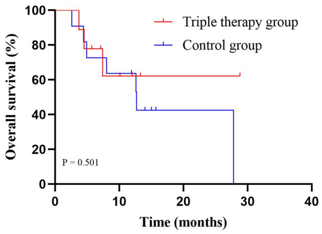

No. at risk					
Triple therapy group	9	4	1	0	0
Control group	11	7	1	0	0

C
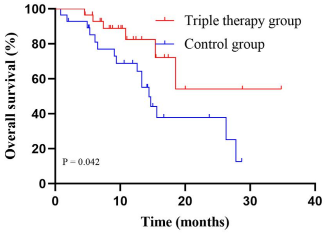

No. at risk					
Triple therapy group	29	16	3	1	0
Control group	28	17	4	0	0

D
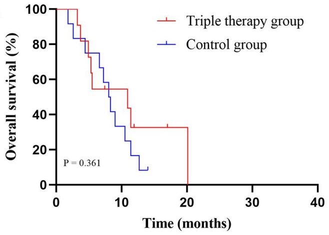

No. at risk					
Triple therapy group	11	5	1	0	0
Control group	12	4	0	0	0

E
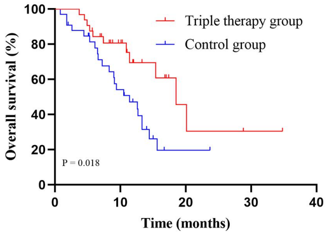

No. at risk					
Triple therapy group	32	17	3	1	0
Control group	33	16	1	0	0

F
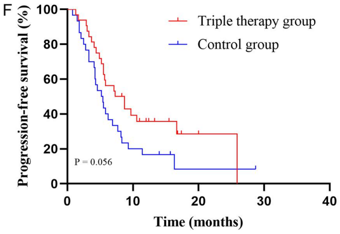

No. at risk					
Triple therapy group	32	11	2	0	0
Control group	30	6	1	0	0

G
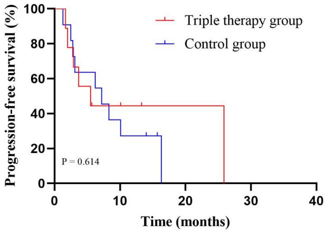

No. at risk					
Triple therapy group	9	3	1	0	0
Control group	11	4	0	0	0

H
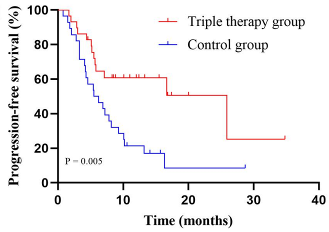

No. at risk					
Triple therapy group	29	12	3	1	0
Control group	28	8	1	0	0

I
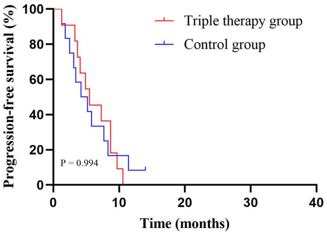

No. at risk					
Triple therapy group	11	1	0	0	0
Control group	12	2	0	0	0

J
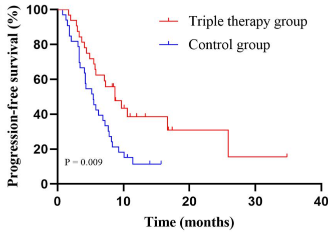

No. at risk					
Triple therapy group	32	10	2	1	0
Control group	33	6	0	0	0
